# Chinese Herbal Medicine for Postpartum Depression: A Systematic Review of Randomized Controlled Trials

**DOI:** 10.1155/2016/5284234

**Published:** 2016-09-28

**Authors:** Yongle Li, Zijie Chen, Ning Yu, Keyu Yao, Yiwen Che, Yupeng Xi, Shuangqing Zhai

**Affiliations:** ^1^School of Preclinical Medicine, Beijing University of Chinese Medicine, Beijing 100029, China; ^2^College of Traditional Chinese Medicine, Inner Mongolia Medical University, Hohhot 010110, China; ^3^Eye Hospital, China Academy of Chinese Medical Sciences, Beijing 100040, China

## Abstract

*Background*. Postpartum depression (PPD) does great harm to women following childbirth. The aim of this study was to conduct a systematic review of the literature to assess the efficacy and safety of CHM for the treatment of PPD.* Methods*. Published or ongoing registered trials were searched for from the inception of the various databases to December 31, 2015. Data extraction and methodology assessment were conducted independently by two researchers. RevMan 5.3 software was used to analyze the data.* Results*. Forty-seven registered clinical trials (RCTs) were identified and reviewed. The results showed CHM alone or in combination with routine treatments could reduce HAMD score, EPDS score, incidence of adverse events, TESS, and SERS. CHM combined with routine treatment was more effective in increasing serum estradiol levels and reducing progesterone levels than routine treatment alone. Meanwhile, pooled data revealed that MRLQS combined with routine treatments or MRLQS plus MSHS combined with routine treatments were more effective than other therapeutic methods in TCM. MRLQS plus MSHS alone was found to be an effective alternative when compared to routine treatments.* Conclusions*. This review suggested that CHM was safe and effective in the treatment of PPD. However, this could not be proven conclusively. To ensure evidence-based clinical practice, more rigorously designed trials are warranted.

## 1. Background

Childbirth is an important experience for women, during which great physical and psychological changes can occur. Postpartum depression (PPD) is a common complication of childbirth, mainly affecting a woman's mentality during the first postpartum year [1]. It is accompanied by changes in thought and behavior and physical symptoms as well. It may negatively affect a woman's quality of life, limit her capacity to fulfill her maternal role, impair mother-infant bonding, and strain relationship with her partner. It even has negative influences on the child's emotional, cognitive, social, and behavioral developments [2–5]. The prevalence of PPD was estimated to be much greater than 10%–15% [1]. Some studies have reported that 60% or more of mothers of newborns were affected in certain areas [6, 7].

The etiology of PPD is multifactorial. Psychological and sociological changes are important risk factors. A personal and family history of depression, poor marital relationship, poor social support, stressful life events, low socioeconomic status, unplanned/unwanted pregnancy, and regional culture may increase the risk for this disease [8–10]. In addition, the reported trigger for the development of PPD refers to biological changes, such as estrogen and progesterone balance, hypothalamic-pituitary-adrenal axis, serotonergic neurotransmitter system, and certain genetic variations [11–15].

Due to differing causative factors, the treatments for PPD are diverse. Selective serotonin reuptake inhibitors (SSRIs) and serotonin-norepinephrine reuptake inhibitors (SNRIs) are antidepressants used as first-line agents. Hormone treatments are considered to be an alternative method of pharmacotherapy. Psychotherapeutic and psychosocial interventions are the important nonpharmacotherapeutic methods used in clinics. Although various therapeutic approaches are available, sufficient evidence is not available to justify the use of any one of these approaches for PPD in clinical practice [1, 16–19].

Chinese herbal medicine (CHM), which is the most important component of traditional Chinese medicine (TCM), is widely used in China and is increasingly being used worldwide. According to TCM theory, the etiology of PPD mainly includes stagnation of the liver, stasis of the blood, deficiency of liver blood, phlegm-dampness, deficiency of kidney yin, deficiency of heart yin and blood, and deficiency in the heart and spleen [20–26]. The clinical treatments of TCM should conduct syndrome differentiation (bian zheng) and project corresponding therapeutic methods to prescription Chinese medicine formula according to analysis of etiology and syndrome. Many clinical trials involving CHM for the treatment of PPD have been conducted and report promising results. Although a meta-analysis (involving 9 trials and 716 patients) based on integrated traditional Chinese and Western medicine for the treatment of PPD has been reported in a Chinese journal [27], its applicability is limited due to its focus on integrative medicine, and only the overall response rate was analyzed in the literature. A comprehensive summary of the safety and efficacy of CHM for the treatment of PPD does not currently exist. Therefore, we systematically reviewed the literature to assess this aspect and to justify the use of CHM for the treatment of PPD in clinical practice.

## 2. Methods

The protocol used for this systematic review was published in the PROSPERO database (http://www.crd.york.ac.uk/PROSPERO/display_record.asp?ID=CRD42014013258).

### 2.1. Eligibility Criteria

Randomized controlled trials (RCTs) testing the effectiveness of CHM in the treatment of PPD in women were included. There was no limitation on language, blinding, or publication format. Quasi-randomized trials were excluded. A clear description of the diagnostic criteria used must have been included. The patients included in this review were at least 16 years of age. CHM included the use of a single herb, Chinese patent medicine, practitioner-prescribed herbal formula, or products extracted from natural herbs. There was no limitation on dosage, administration regimen, or duration of treatment. The control could be a placebo, no treatment, or routine treatments. Trials evaluating the use of CHM combined with routine treatments versus the routine treatments alone were also included. The primary outcome measured was improvement in depression at the end of treatment or at the time of follow-up. Valid assessment tools included the Hamilton Depression Rating Scale (HAMD), Edinburgh Postnatal Depression Score (EPDS), Self-Rating Depression Scale (SDS), Beck Depression Inventory (BDI), Postpartum Depression Screening Scale (PDSS), and adverse events associated with CHM. Additionally, some studies measured changes in the levels of estradiol and progesterone.

### 2.2. Search Strategy and Study Selection

Two authors (Zijie Chen and Ning Yu) conducted a systematic search for published, unpublished, and ongoing trials. The Cochrane Central Register of Controlled Trials (CENTRAL), PubMed, Excerpta Medica database (EMBASE), Chinese Biomedical Literature Database (CBM), Chinese National Knowledge Infrastructure Database (CNKI), Chinese Science and Technology Periodical Database (VIP), and Wan Fang Database were searched from the date of their inception until December 31, 2015. The World Health Organization (WHO) International Clinical Trial Registry Platform (ICTRP) portal and the website of International Clinical Trial Registry by US National Institutes of Health (http://clinicaltrials.gov/) were searched for ongoing registered clinical trials.

The following search terms were used individually or combined: “maternal depression”, “post partum depression”, “postpartum depression”, “puerperium depression”, “postnatal depression”, “Chinese traditional”, “Chinese herb”, “Oriental traditional”, “herb”, “herbal medicine”, and “random”. There were no restrictions on language, publication year, and publication status. The details were shown in Supplementary Table  1 in Supplementary Material available online at http://dx.doi.org/10.1155/2016/5284234.

Titles and abstracts were screened to identify trials that potentially met the criteria of this study. The full texts of selected trials were reviewed by two authors (Zijie Chen and Ning Yu) to determine eligibility for inclusion. The reference lists for all eligible articles were perused to collect additional trials. Any discrepancies were resolved by consensus and discussion with a third party (Shuangqing Zhai) if necessary.

### 2.3. Data Extraction

Data information was extracted independently by two authors (Keyu Yao and Yupeng Xi). A predesigned structured data extraction form was used for the eligible trials, which mainly included publication year, study type, random sequence generation, allocation concealment, blinding, diagnostic criteria, sample size, baseline characteristics of the participants, type of syndrome differentiation, therapeutic methods of TCM, intervention, control, outcomes, follow-up, and adverse events. The authors of the trials were contacted if any information was unclear or missing. Disagreements were resolved by discussion and consensus was reached with the help of a third party (Shuangqing Zhai).

### 2.4. Assessment of Risk of Bias

The risk of bias in eligible trials was assessed independently by two authors (Keyu Yao and Yupeng Xi) according to the criteria described in the Cochrane Handbook version 5.1.0 [73]. The criteria mainly included random method, allocation concealment, blinding of participants and personnel, blinding of outcome assessment, incomplete outcome data, selective reporting, and other sources of bias. Each trial was classified as having a low, unclear, or high risk of bias. Any discrepancies were settled by discussion with a third party (Shuangqing Zhai). If necessary, the author of the trial was contacted to clarify any ambiguities.

### 2.5. Data Analysis

RevMan 5.3 software was used to analyze the data. If the data were available, the following comparisons were performed: CHM versus no treatments, CHM versus placebo, and CHM with or without routine treatments versus routine treatments alone. The diversity of therapeutic methods used in TCM may be an important reason for the high heterogeneity in the interventions, and the subgroups were further analyzed in this review. A meta-analysis was performed if homogeneity in study design, participants, intervention, control, and outcome measures was acceptable. A random-effect model was used for the meta-analysis if the trials had significant heterogeneity (*I* ^2^ > 50%) [74]. Otherwise, a fixed-effect model was used. The effect estimate was expressed as risk ratio (RR) with a 95% confidence interval (CI) for categorical data and as mean difference (MD) with a 95% CI for continuous data. Funnel plots were generated to detect publication bias when more than ten trials were identified [74].

## 3. Results

### 3.1. Description of Studies

**Figure 1:**
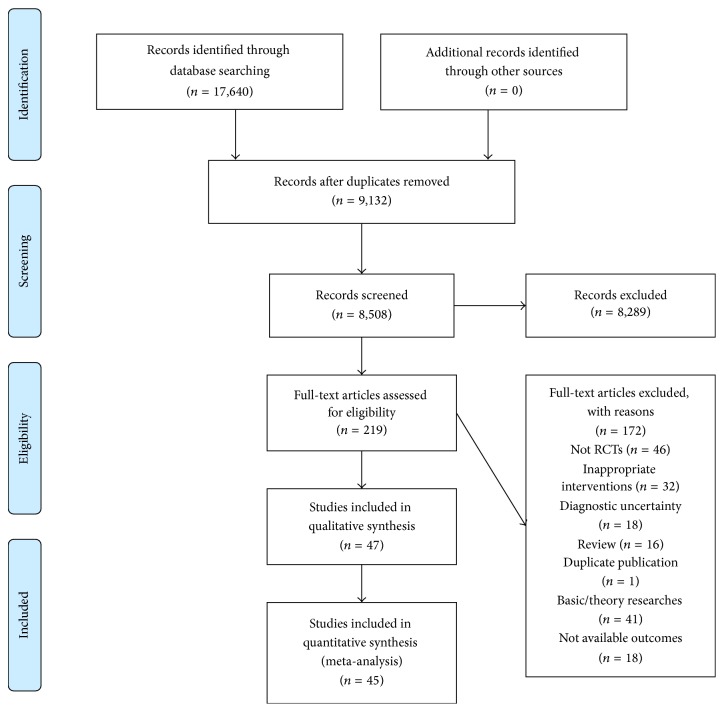
Flow diagram of the search process and study selection for the systematic review.

The initial search yielded 17,640 records from seven electronic databases: CNKI (*n* = 2,237), CBM (*n* = 4,302), VIP (*n* = 5,245), Wan Fang (*n* = 5,853), PubMed (*n* = 3), CENTRAL (*n* = 0), and EMBASE (*n* = 0). After removing 9,132 records, which were duplicates from the different databases, 8,508 citations were screened. 8,289 records were excluded for various reasons determined while reading the titles and abstracts. Full text of the remaining 219 articles was retrieved and assessed in detail for eligibility. An additional 172 trials were excluded due to inappropriate interventions, diagnostic uncertainty, basic/theory researches, review, duplicate publication, absence of randomized controlled trials, or nonavailability of outcomes. Ultimately, 47 RCT articles [21, 26, 28–72] met the inclusion criteria and were included in this systematic review. A flow chart detailing the search process and study selection is displayed in Figure 1. We also searched trial registries that identified only one ongoing clinical trial (http://clinicaltrials.gov/). However the trial (ClinicalTrials.gov Identifier: NCT01178008) focused on acupuncture for the treatment of PPD and did not meet our criteria for inclusion.

All 47 trials were conducted and published in China; 44 were journal articles (93.62%) [21, 26, 28–46, 48–53, 55, 56, 58–72] and 3 were postgraduate papers (6.38%) [47, 54, 57]. All of the trials included two arms with the exception of one [63]. That trial included four arms. The main characteristics of the included trials are displayed in Table 1. A total of 3,795 participants (1,959 in intervention groups and 1,836 in control groups) with PPD and ranging in age from 18 to 43 years old [41, 48] were included. The sample sizes ranged from 45 to 150 [42, 62]. Baseline demographics were provided in all of the studies except one [69], and all reported no significant differences between the intervention and control groups. To obtain the diagnostic criteria for PPD, 36 trials used DSM-IV [21, 26, 30–32, 34–38, 40, 42–45, 47, 48, 50–59, 61–63, 66–68, 70–72], 9 trials used CCMD-III [28, 29, 33, 39, 46, 49, 60, 65, 69], one trial used ICD-X [64], and one trial used both of CCMD-III and DSM-V [41]. Nine trials provided information on the patients' syndrome differentiation (bianzheng fenxing) [28, 46–48, 57, 58, 60, 63, 68], and 18 trials [26, 52, 54–57, 61, 63, 67, 68, 72] provided TCM therapeutic methods used to formulate individualized herbal preparations.

There were 2 trials [57, 63] that evaluated CHM versus a placebo, 9 trials [34, 35, 42, 50, 62, 64, 65, 71, 72] that evaluated CHM versus routine treatments, and 36 trials [21, 26, 28–33, 36–41, 43–49, 51–56, 58–61, 66–70] that evaluated CHM plus routine treatments versus routine treatments alone. 39 different types of CHM were investigated. The specific compositions of CHM are shown in Table 2. All types of CHM could be categorized into eight categories according to the therapeutic methods of TCM, as is shown in Table 2. The routine treatments mainly consisted of SSRIs (e.g., fluoxetine hydrochloride, paroxetine hydrochloride), SNRIs (e.g., citalopram, duloxetine, venlafaxine, sertraline), TCAs (e.g., amitriptyline), and psychotherapeutic and psychosocial interventions. The total treatments duration varied from 4 weeks to 6 months [47, 62].

All trials included primary outcomes and secondary outcomes. 34 trials [21, 28, 31, 35–42, 44–50, 52, 54, 55, 57, 58, 60, 61, 64–72] reported depression scores measured by HAMD or EPDS. Four trials [47, 57, 59, 63] reported changes in estradiol and progesterone concentrations. 39 trials [21, 26, 29, 30, 32–39, 41–44, 46, 49–57, 59–69, 71, 72] reported adverse events. Among these, 30 trials [26, 29–32, 34–36, 41–43, 46, 49–55, 57, 59, 60, 62–66, 69, 71, 72] reported incidence of adverse events, 6 trials [33, 37, 39, 61, 64, 69] used Treatment Emergent Symptoms Scale (TESS), and 2 trials [38, 44] used Rating Scale for Side Effects (SERS) to assess the safety of CHM for the treatment of PPD. Only 3 trials [32, 43, 49] conducted follow-up examinations after the end of treatment.

### 3.2. Methodological Quality

**Figure 2:**
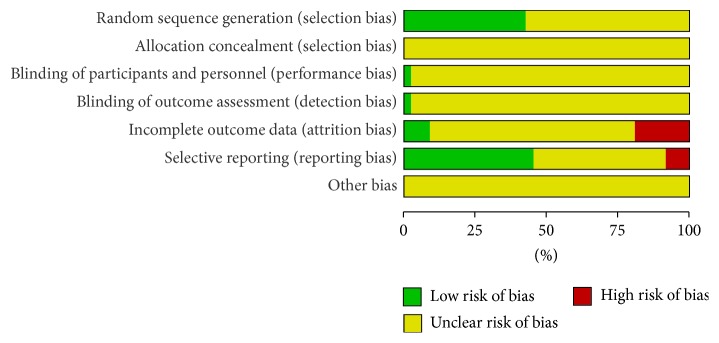
Risk of bias graph: review authors' judgments about each risk of bias item presented as percentages across all included studies.

All the trials provided limited information about design and methodology. The randomized allocation of participants among the groups was mentioned in all trials, but only 20 trials [21, 26, 30, 33, 35, 38, 41, 45, 49, 50, 52, 54, 57, 60, 63, 65, 67, 68, 70, 71] described the specific methods used for sequence generation. These included random number table, drawing of lots, and randomized card law. Allocation concealment was only reported in one trial [57]; however it could not be judged whether this was properly conducted because of insufficient information. Only one trial [63] was of double-blind design, and the rest of trials did not provide any information regarding blinding. Information detailing withdrawal from the study was provided in 13 trials [29, 37, 39, 43, 45, 47, 52, 54, 57, 63, 67, 68, 72]; of these, 3 trials [45, 47, 67] reported that no participants dropped out, 3 trials [52, 57, 68] provided withdrawal reasons, and only one trial [57] conducted intention-to-treat analysis. To assess selective reporting, we searched the included registration information using trial registries. Registration information and the protocol used for the study were not accessible for all trials. Therefore, we assessed selective reporting by comparing the outcome measures mentioned in materials and methods section with the outcomes reported in the results. 20 trials [26, 28, 33, 36, 38, 41, 42, 44, 47, 48, 50, 54, 57, 58, 60, 62, 64, 67, 68, 72] were evaluated as low risk because all of the reported outcomes were described in the methods, 4 trials [45, 46, 63, 66] were evaluated as high risk because the outcomes reported in the results did not match prespecified outcomes, and 23 trials [29–32, 34, 35, 37, 39, 40, 43, 49, 51–56, 59, 61, 65, 67, 69–71] were evaluated as unclear because no description of outcomes was provided in the materials and methods section. Only one trial [68] conducted a pretrial estimation of required sample size. Consequently, all of the trials had a high risk of bias and were assessed to be of low quality (Figure 2 and Supplementary Figure  1).

### 3.3. Effect Estimates

Among the 47 included trials, the possibility of pooling the effect estimates was limited due to differences in the methods of intervention used. Therefore, the findings were reported under three categories including CHM versus placebo (2 trials), CHM versus routine treatments (9 trials), and CHM plus routine treatments versus routine treatments alone (36 trials). Additionally, TCM therapeutic methods were further studied by subgroup analysis. Although few trials described the therapeutic methods of TCM used, they could be deduced by CHM component. The deductions were performed by two authors (Yongle Li and Yiwen Che), and any disagreements were resolved with the help of a third party (Shuangqing Zhai). The therapeutic methods were divided into the method of relieving liver qi stagnation (MRLQS) (shu gan jie yu fa), the method of strengthening heart and spleen (MSHS) (bu yi xin pi fa), the method of blood-activating and stasis-dissolving (MBASD) (huo xue hua yu fa), the method of tonifying qi and blood (MTQB) (bu yi qi xue fa), or a combination of the methods to illustrate the existence of heterogeneity in the subgroups.

#### 3.3.1. HAMD Scores

30 trials [21, 28, 31, 35–40, 42, 44–47, 49, 50, 52, 54, 55, 58, 60, 61, 64–67, 69–72] reported outcomes in HAMD score after treatment. Seven trials [35, 42, 50, 64, 65, 71, 72] which compared CHM with routine treatments showed statistical heterogeneity in the consistency of the results (*I* ^2^ = 95%), and a random effects model was used for meta-analysis. The pooled results showed that there were statistically significant differences between CHM and routine treatments (MD −2.60, 95% CI −4.55 to −0.64) in reducing HAMD score. Subgroup analysis showed that MRLQS plus MSHS (4 trials) [35, 50, 64, 65] had a superior effect to routine treatments (MD −1.91, 95% CI −3.37 to −0.44). MRLQS plus MTQB (1 trial) [71] and MRLQS plus MSHS plus MBASD (1 trial) [42] had a statistically significant reduction in HAMD score as compared to routine treatments. Meanwhile, one trial [72] reported no significant difference between MRLQS plus MTQB plus MBASD and routine treatments. To compare the effects of CHM plus routine treatments with routine treatments alone, 23 trials [21, 28, 31, 36–40, 44–47, 49, 52, 54, 55, 58, 60, 61, 66, 67, 69, 70] were evaluated. Heterogeneity testing of these trials indicated a significant difference (*I* ^2^ = 85%); therefore, a random effects model was used. The pooled results showed that there were statistically significant differences between CHM plus routine treatments and routine treatments alone (MD −3.00, 95% CI −3.73 to −2.26) in reducing HAMD scores. Subgroup analysis was conducted on the Chinese medicine therapeutic methods of MRLQS, MTQB, MSHS, MRLQS plus MSHS, MRLQS plus MBASD, and MRLQS plus MTQB. Pooled results from 13 trials [21, 31, 37, 38, 44, 45, 49, 52, 55, 58, 60, 61, 67] showed that MRLQS combined with routine treatments had superior effectiveness as compared to routine treatments alone (MD −3.19, 95% CI −4.41 to −1.97) in reducing HAMD score. Three trials [39, 46, 66] indicated that MTQB combined with routine treatments was more effective than routine treatments alone (MD −2.67, 95% CI −4.48 to −0.87). Four trials [28, 36, 40, 70] demonstrated that MRLQS plus MSHS combined with routine treatments had superior effectiveness as compared to routine treatments alone (MD −2.67, 95% CI −3.20 to −2.14). As shown in Table 3, the results demonstrated that MSHS combined with routine treatments (1 trial) [47] or MRLQS plus MBASD combined with routine treatments (1 trial) [54] was more effective than routine treatments alone in reducing HAMD score. One trial [69] demonstrated no statistically significant differences between MRLQS plus MTQB combined with routine treatments and routine treatments alone (shown in Table 3).

#### 3.3.2. EPDS Scores

Six trials [28, 41, 42, 48, 57, 68] provided data on EPDS score changes after treatment. Only one trial [57] compared CHM using MSHS with a placebo. The study results showed that MSHS was more effective than the placebo (MD −2.67, 95% CI −3.88 to −1.46) in reducing EPDS score. Two trials [42, 68] which compared CHM with routine treatments conducted a random effects model for meta-analysis because of statistical heterogeneity of the results (*I* ^2^ = 90%). The pooled results showed significant beneficial effects of CHM as compared to routine treatments (MD −3.36, 95% CI −6.05 to −0.66). Subgroup analysis in these trials revealed that the use of MTQB plus MBASD (1 trial) [68] or MRLQS plus MSHS plus MBASD (1 trial) [42] resulted in a statistically significant reduction in EPDS score when compared with routine treatments alone. Three trials [28, 41, 48] compared CHM combined with routine treatments with routine treatments alone. These trials used MRLQS plus MSHS as the therapeutic approaches of CHM and used a random effects model for pooled analysis because of statistical heterogeneity (*I* ^2^ = 75%). The pooled result showed that CHM (MRLQS plus MSHS) combined with routine treatments had superior effectiveness when compared to routine treatments alone (MD −3.80, 95% CI −5.27 to −2.34) in reducing EPDS score (shown in Table 3).

#### 3.3.3. Serum Estradiol

Four trials [47, 57, 59, 63] reported the effect on serum estradiol of CHM alone or in combination with routine treatments. Two trials [57, 63] compared the effect of CHM with a placebo and used a random effects model for meta-analysis because of statistical heterogeneity (*I* ^2^ = 88%). The pooled result showed no statistically significant differences between the groups (MD 29.01, 95% CI −2.54 to 60.56) in increasing serum estradiol. Subgroup analysis showed that MRLQS plus MTQB (1 trial) [63] was more effective than routine treatments alone. No significant difference was observed in another trial [57] that compared MSHS with a placebo. In comparing CHM combined with routine treatments to routine treatments alone, the data from 2 trials [47, 59] was pooled and a fixed effects model was used because of no statistical heterogeneity (*I* ^2^ = 11%). The results revealed that CHM combined with routine treatments had superior effectiveness when compared with routine treatments alone (MD 38.36, 95% CI 35.38 to 41.33). The subgroup analysis revealed that MSHS combined with routine treatments (1 trial) [47] or MTQB combined with routine treatments (1 trial) [59] was more effective than routine treatments alone in increasing serum estradiol (shown in Table 3).

#### 3.3.4. Progesterone

Four trials [47, 57, 59, 63] reported progesterone levels in patients receiving CHM alone or in combination with routine treatments. Two trials [57, 63] compared effect of CHM to a placebo, and the data was pooled with a fixed effects model being used because of no statistical heterogeneity (*I* ^2^ = 0%). The results showed that CHM was more effective than a placebo in decreasing progesterone (MD −2.11, 95% CI −3.08 to −1.14). The subgroup analysis showed that MRLQS plus MTQB (1 trial) [63] was more effective than routine treatments alone, and no significant differences were observed in another trial [57] when MSHS was compared to a placebo. In comparing CHM combined with routine treatments to routine treatments alone, a random effects model was used for two pooled trials [47, 59] because of statistical heterogeneity (*I* ^2^ = 88%). The results showed that CHM combined with routine treatments was more effective than routine treatments alone (MD −8.14, 95% CI −15.70 to −0.58). The subgroup analysis showed that MTQB combined with routine treatments (1 trial) [59] resulted in a statistically significant reduction in progesterone as compared to routine treatments alone. There were no significant differences observed in another trial [47] when MSHS was compared with a placebo (shown in Table 3).

#### 3.3.5. Adverse Events

In this review, 8 trials [21, 28, 40, 45, 47, 48, 58, 70] did not report any information regarding adverse events, and 39 trials [26, 29–39, 41–44, 46, 49–57, 59–69, 71, 72] mentioned adverse events. In trials comparing CHM with a placebo, one trial [57] reported no adverse events occurring in the intervention group. The placebo group, however, reported adverse events including dryness of mouth and constipation. One trial [63] reported the adverse event of mouth dryness in the CHM group. In trials comparing CHM with routine treatments alone, 5 trials [42, 50, 62, 64, 65] reported adverse events occurring in both groups, and 4 trials [34, 35, 71, 72] reported no adverse events occurring in the CHM groups. Adverse events occurring in the CHM groups included gastric discomfort, nausea, dizziness, sleepiness, and constipation. In trials comparing CHM combined with routine treatments to routine treatments alone, 24 trials [26, 30–33, 36–39, 41, 43, 44, 46, 49, 51, 53–55, 59–61, 66, 67, 69] reported adverse events occurring in both groups. Two trials [29, 52] reported no adverse events occurring in CHM combined with routine treatments groups, and 2 trials [56, 68] reported no adverse events occurring in both groups. The adverse events occurring in the CHM combined with routine treatments group included loss of appetite, sleep disorders, nausea, vomiting, constipation, tachycardia, hypodynamia, dysphoria, tremor, dizziness, diarrhea, sleepiness, insomnia, headache, weight gain, hypotension, akathisia, blurred vision, chest distress, heart palpitations, skin rash, hyperhidrosis, abdominal discomfort, urination disorders, sexual dysfunction, transaminase elevation, blood system involvement, abnormal liver function, and abnormal ECG. 30 trials [26, 29–32, 34–36, 41–43, 46, 49–55, 57, 59, 60, 62–66, 69, 71, 72] reported a number of participants who experienced adverse events, 6 trials [26, 33, 37, 39, 61, 64] utilized TESS, and 2 trials [38, 44] utilized SERS to evaluate the adverse events. For better evaluation of the safety of TCM in the treatment of PPD, related data were analyzed.

According to a meta-analysis of the incidence of adverse events, 2 trials [57, 63] comparing CHM with a placebo warranted a fixed effects model for pooling analysis due to no statistical heterogeneity (*I* ^2^ = 33%). The results revealed no significant differences between the two groups in reducing the incidence of adverse events (RR 0.67, 95% CI 0.11 to 3.91). Subgroup analysis revealed no significant differences between MSHS (1 trial) [57] or MRLQS plus MTQB (1 trial) [63] and a placebo. For the 9 trials comparing CHM with routine treatments alone [30, 34, 42, 50, 62, 64, 65, 71, 72] data was pooled and a random effects model was used because of statistical heterogeneity (*I* ^2^ = 57%). The results showed that CHM had superior effectiveness to routine treatments alone in reducing the incidence of adverse events (RR 0.18, 95% CI 0.09 to 0.38). Subgroup analysis showed that MTQB (2 trial) [34, 62] was more effective than routine treatments alone (RR 0.12, 95% CI 0.04 to 0.36). MRLQS plus MSHS (3 trial) [35, 50, 64, 65] was more effective than routine treatments alone (RR 0.21, 95% CI 0.10 to 0.47). MRLQS plus MTQB (1 trial) [71] or MRLQS plus MTQB plus MBASD (1 trial) [72] was more effective than routine treatments alone. No significant difference was observed when MRLQS plus MSHS plus MBASD (1 trial) [42] was compared with routine treatments alone. For the 19 trials [30–33, 36, 41, 43, 44, 46, 49, 51–55, 59, 60, 66, 68, 69] comparing CHM combined with routine treatments to routine treatments alone, a random effects model was used to pool data because of statistical heterogeneity (*I* ^2^ = 56%). The results revealed that CHM combined with routine treatments had superior effectiveness to routine treatments alone in reducing the incidence of adverse events (RR 0.49, 95% CI 0.37 to 0.65). Subgroup analysis of 9 trials [30, 31, 43, 44, 49, 51, 52, 55, 60] utilizing MRLQS combined with routine treatments showed that this treatment had superior effectiveness to routine treatments alone (RR 0.45, 95% CI 0.32 to 0.64). Three trials [36, 41, 53] utilizing MRLQS plus MSHS combined with routine treatments showed that this treatment was more effective than routine treatments alone (RR 0.45, 95% CI 0.22 to 0.93). Three trials [46, 59, 66] involving MTQB combined with routine treatments, 2 trials [32, 69] involving MRLQS plus MTQB combined with routine treatments, and 2 trials [54, 68] involving MRLQS plus MBASD combined with routine treatments demonstrated no significant differences when compared to routine treatments alone (shown in Table 3).

Meanwhile, one trial [64] utilized TESS to compare MRLQS plus MSHS with routine treatments alone. The results showed that MRLQS plus MSHS was more effective than routine treatments alone. Five trials [33, 37, 39, 61, 69] compared CHM combined with routine treatments to routine treatments alone utilizing TESS. A random effects model was used to pool data because of statistical heterogeneity (*I* ^2^ = 71%). The results showed that CHM combined with routine treatments had superior effectiveness to routine treatments alone in reducing TESS (MD −1.80, 95% CI −2.46 to −1.14). Subgroup analysis of two trials [37, 61] showed that MRLQS combined with routine treatments had superior effectiveness to routine treatments alone (MD −2.31, 95% CI −3.12 to −1.50). MTQB combined with routine treatments (1 trial) [39], MRLQS plus MTQB combined with routine treatments (1 trial) [69], and MRLQS plus MBASD combined with routine treatments (1 trial) [33] were all shown to be more effective than routine treatments alone. A pooled analysis of 2 trials [38, 44] revealed that MRLQS combined with routine treatments had superior effectiveness to routine treatments alone in reducing SERS (MD −5.19, 95% CI −9.73 to −0.64) (shown in Table 3).

#### 3.3.6. Publication Bias Assessment

**Figure 3:**
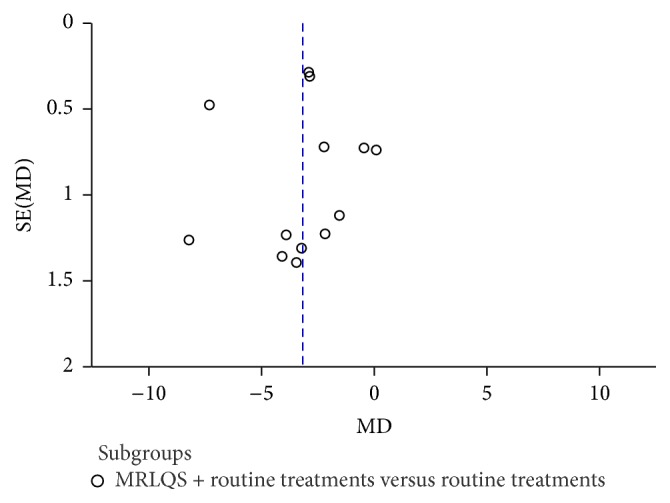
Funnel plot of comparison: MRLQS combined with routine treatments versus routine treatments alone; outcome: HAMD scores.

A funnel plot for HAMD score following treatment with MRLQS combined with routine treatments in the experimental group and routine treatments alone in the control group was conducted to investigate the publication bias. The result indicated that publication bias may have existed in this review (Figure 3).

## 4. Discussion

### 4.1. Principal Findings

PPD has become a public health problem concern, as it is harmful to postpartum women and their families. Women have an important responsibility in the parenting of newborns. Therefore, selecting a safe and effective method of treatment is of particular importance. This study conducted a meta-analysis to assess the safety and efficacy of treatments for PPD using the therapeutic methods of TCM.

This review evaluated 47 trials and the pooled results demonstrated that CHM treatments alone or in combination with routine treatments were effective in decreasing HAMD scores, EPDS scores, and incidence of adverse events. It indicated that CHM may be a safer and more effective treatment for PPD than routine treatments alone. A subgroup analysis was also conducted because the diversity of therapeutic methods of TCM used was an important factor influencing clinical effect. HAMD score was considered to be an important tool for evaluating the clinical efficacy of PPD. CHM plus routine treatments (76.60%, 36/47) was the most common treatment approach. According to pooled data from related trials, the results suggest that MRLQS combined with routine treatments is more effective than MRLQS plus MSHS combined with routine treatments or MTQB combined with routine treatments in reducing HAMD score when each is compared with routine treatments alone. In this review, we also observed that MRLQS plus MSHS alone had a better clinical efficacy than routine treatments alone for PPD. However, it was difficult to evaluate other therapeutic methods of TCM individually or in combination with routine treatments because the data could not be pooled for analysis.

In the TCM theory, depression belongs to the category of emotion and will diseases. It is mainly caused by emotional triggers and viscera weakness. Stagnation of liver-qi caused by multiple etiologies is the most important mechanism of depression because of the role of the liver in regulating emotion. Meanwhile, postpartum women had the physiological characteristics of deficiency syndrome and stasis syndrome according to the TCM theory [68]. In the case of maternal frail state, excessive thinking exhausts the heart blood and spleen qi, which leads to deficiencies in both heart and spleen. Hypofunction of heart and spleen leads to mind (xin shen) dystrophy and causes or aggravates the symptoms of PPD. Stagnation of liver qi and deficiency of both heart and spleen are the principal pathogenic mechanisms of PPD. Therefore, stagnation of liver qi alone or in combination with deficiencies of both heart and spleen is the main type of syndrome differentiation (bian zheng fen xing) according to TCM theory. MRLQS plus MSHS is a potential treatment option besides MRLQS alone for the management of PPD. This matches the principal pathogenesis and syndrome differentiation according to Chinese medicine. This study found that MRLQS plus MSHS in combination with routine treatments was clinically effective when compared with routine treatments alone in reducing HAMD score. Meanwhile, mean difference (MD) value of MRLQS plus MSHS in combination with routine treatments was greater than MRLQS plus MSHS alone, which suggests that a combination of MRLQS plus MSHS with routine treatments may have synergistic effects. Meta-analysis of EPDS further confirmed the effectiveness of MRLQS plus MSHS in combination with routine treatments. In terms of safety, MRLQS in combination with routine treatments or MRLQS plus MSHS in combination with routine treatments was associated with fewer adverse effects than routine treatments alone according to the existing data. Therefore, based on our analysis of clinical research and TCM theory, the use of MRLQS in combination with routine treatments or MRLQS plus MSHS in combination with routine treatments appears to be a superior approach for the treatment of PPD. Additionally, because MRLQS plus MSHS alone is safer and more effective than routine treatments, MRLQS plus MSHS might be an alternative option for patients who cannot tolerate antidepressant drugs.

In the present review, 47.22% of the trials (17/36) utilized MRLQS in combination with routine treatments for the management of PPD. Xiao yao powder (11/17) was identified as the most frequently used Chinese herbal compound in related studies. A prior systematic review revealed the effectiveness of Xiao yao powder in the treatment of depression [75]; further, it indicated that Xiao yao powder may be effective in the treatment of PPD. In trials evaluating MRLQS plus MSHS, shugan jieyu capsule was the most frequently used Chinese herbal compound, and a prior systematic review also reported evidence supporting the effectiveness of shugan jieyu capsule for the treatment of depression [76]. Although the present review confirmed the effectiveness of MRLQS or MRLQS plus MSHS and their corresponding Chinese herbal compounds, this information should be used cautiously. The therapeutic method of TCM and its corresponding Chinese herbal compound should be selected based on Chinese medicine syndrome differentiation (bian zheng). Due to the high risk of bias, the significant clinical heterogeneity observed between the trials, and the relatively small sample size, a definite conclusion still cannot be drawn.

### 4.2. Limitations of This Review

There were some limitations in this review which contributed to the inconclusive results. First, all trials were of poor methodological quality, and there was a high risk of bias. Of the 47 trials, only 42.55% (20/47) provided information on the methods for sequence generation, and most of them provided insufficient information to ascertain whether the randomization was conducted properly. The remainder of the trials only mentioned that “the patients were randomized into two groups.” Allocation concealment was mentioned in only one trial [57]. Although the trial indicated that the random numbers had been placed in a sealed envelope, it was not mentioned whether the envelope was translucent. Only one trial [63] claimed double-blind design and presample size estimation. The majority of the trials (72.34%) did not provide any information regarding withdrawal from the study, and only one trial [57] conducted intention-to-treat analysis.

Second, the therapeutic methods of TCM and Chinese herbal compounds investigated may be responsible for the considerable heterogeneity in this review. In the practice of TCM, the herbalist should evaluate the patients symptoms to conduct syndrome differentiation and then propose the corresponding therapeutic methods of TCM. The herbal formulation is prescribed based on the therapeutic methods of TCM. This is called syndrome differentiation and treatment (bian zheng lun zhi), which is the strength of TCM. According to TCM theory, one type of syndrome differentiation should match one type of fixed therapeutic method of TCM. Different types of Chinese herbal formulations are prescribed to create individualized treatment plans for the patients under the guidance of the relatively fixed therapeutic methods of TCM. Although the specifics of CHM may differ among the trials, the therapeutic methods are consistent. Therefore, the therapeutic methods of TCM are at the core of CHM treatment. However, only 19.15% of the trials (9/47) provided information on the patients' type of syndrome differentiation, only 38.30% of the trials (18/47) provided information on the therapeutic methods of TCM, and only 12.77% of the trials (6/47) contained both. Meanwhile, 39 different Chinese herbs were investigated in this review and could be divided into 8 categories of therapeutic methods of TCM. The formulations included herbal formulas (27 trials), powder (2 trials), granules (2 trials), pills (6 trials), capsules (5 trials), herbal extracts (3 trials), and liquids (2 trials). Therefore, it was difficult to draw a definite conclusion regarding which therapeutic method of TCM or Chinese herbal compound was the most effective.

Third, the majority of the trials did not provide follow-up information. Three trials provided follow-up information over a 3–6-month period; however, this is considered insufficient for assessment of the long-term effectiveness of CHM.

Finally, this review found that reporting on adverse events in the included trials was inadequate. No mention of adverse events was made by 21.78% of the trials (10/47). Although 78.72% (37/47) of the trials reported adverse events, the information was limited. No trials reported whether CHM could affect breast-feeding or have adverse effects on newborns. Therefore, the safety of CHM for the treatment of PPD could not be assessed. Deficiencies in this systematic review limit the reliability of the meta-analysis results.

### 4.3. Implications of Further Research

To ensure evidence-based clinical practice, more rigorous multicenter, large, methodological design trials are warranted to provide higher quality evidence. The process of random sequence generation, adequate allocation concealment, double blinding, clear description of reasons for withdrawal, and intention-to-treat should be addressed. CHM should be conducted based on the syndrome differentiation pattern of PPD and the guidelines for TCM therapeutic methods, which have been previously reported. The adverse events associated with CHM should be described. Participants should be followed up for an extended period of time to assess the long-term effects of the treatment. Trials should adhere to CONSORT standards (http://www.consort-statement.org/) to promote internal and external validity.

## 5. Conclusion

In conclusion, the evidence indicates that CHM was effective in treating PPD; additionally, MRLQS combined with routine treatments or MRLQS plus MSHS combined with routine treatments was more effective than routine treatments alone. MRLQS plus MSHS alone might be an effective alternative to routine treatments. However, the evidence detailed in this review should be considered cautiously because of the high risk of bias and the poor methodological quality of the trials. Therefore, rigorously designed trials are needed for further evaluation of the safety and efficacy of CHM for the treatment of PPD.

## Supplementary Material

Details regarding search strategies of databases and risk of bias summary are shown in supplementary material. Search terms used in each database was included in supplementary Table 1. Review authors' judgments about each risk of bias item for included studies were shown in supplementary Figure 1.

## Figures and Tables

**Table 1:** Characteristics of included randomized controlled trials.

Study ID	Diagnosis standard	Sample size I/C	Age (year) I/C	Course of disease I/C	Syndrome differentiation of TCM	Therapeutical method of TCM	Intervention	Control	Duration	Follow-up	Outcome
Cai 2015 [28]	CCMD-III	71/40	32.5 ± 4.5/34.5 ± 2.5	41.6 ± 6.7 d/39.7 ± 5.3 d	Stagnation of liver and la tortura and deficiency of both heart and spleen	Relieving liver qi and invigorating the spleen, tonifying qi and blood.	Shugan jianpi kaixin decoction + sertraline + vitamin D drops + cognitive therapy, behaviour therapy + relaxation therapy + music therapy	Sertraline + vitamin D drops + cognitive therapy, behaviour therapy + relaxation therapy + music therapy	NR	NR	Effective rate, HAMD scores, EPDS scores, SCL-90 scores

Chen et al. 2015 [29]	CCMD-III	30/30	26.1 ± 1.2/25.3 ± 0.4	0.87 ± 0.14 y/0.85 ± 0.16 y	NR	NR	Fuling shenzhi shuangxin pill + sertraline hydrochloride tablets, fluoxetine hydrochloride tables	Sertraline hydrochloride tablets, fluoxetine hydrochloride tables	4 m	NR	Effective rate, adverse events

Chen et al. 2011 [30]	DSM-IV	38/38	30.2 ± 1.5/30.0 ± 1.8	3.8 ± 1.2 m/4.1 ± 1.0 m	NR	NR	Self-made qingyu pingxin decoction + Fluoxetine	Fluoxetine	1 m	NR	Effective rate, adverse events

Ding et al. 2010 [31]	DSM-IV	40/30	27.46 ± 5.215/25.42 ± 4.441	27.15 ± 11.841 d/24.21 ± 9.718 d	NR	NR	Modification decoction of xiaoyao san + Mirtazapine	Mirtazapine	8 w	NR	Obvious effective rate, effective rate, HAMD scores, adverse events

Fan 2011 [32]	DSM-IV	52/52	29.3/28.6	35.5 d/37.7 d	NR	NR	Modification mixture of tianwang buxin decoction + fluoxetine	Fluoxetine	4 w	6 m	Effective rate, adverse events

Fang 2014 [33]	CCMD-III	57/56	26.8 ± 2.9/27.4 ± 3.2	11.2 ± 3.8 w/10.5 ± 3.5 w	NR	NR	Self-made tiaoxue jieyu decoction + venlafaxine hydrochloride sustained-release tablets	Venlafaxine hydrochloride sustained-release tablets	8 w	NR	SAS scores, MADRS scores, adverse events

Gao 2010 [34]	DSM-IV	36/28	25–35/25–35	NR	NR	Tonifying qi and blood, psyche-nourishing and tranquilizing mind.	Modification mixture of yulin decoction	Fluoxetine	6 w	NR	Effective rate, adverse events

Guo et al. 2011 [35]	DSM-IV	38/34	26.14 ± 2.5/25.84 ± 2.7	NR	NR	Strengthening heart and spleen, relieving liver qi stagnation.	Modification decoction of chaihu shugan san and ganmai dazao tang	Fluoxetine	4 w	NR	HAMD scores, effective rate, adverse events

Hao 2015 [36]	DSM-IV	28/28	27 ± 9/27 ± 8	24.5 ± 2.8 w/23.4 ± 3.2 w	NR	NR	Shugan jieyu capsule + citalopram	Citalopram	6 w	NR	HAMD scores, effective rate, adverse events

He and Ren 2008 [37]	DSM-IV	47/44	26.1 ± 2.5/25.8 ± 2.7	NR	NR	NR	Modification decoction of xiaoyao san + fluoxetine	Fluoxetine	4 w	NR	HAMD scores, effective rate, adverse events

Hu 2013 [38]	DSM-IV	50/50	27.6 ± 2.5/26.3 ± 2.7	21 ± 5.4 d/22 ± 5.5 d	NR	NR	Xiaoyao pill + fluoxetine	Fluoxetine	6 w	NR	Effective rate, HAMD scores, adverse events

Jiang et al. 2012 [39]	CCMD-III	35/35	27 ± 5.4/26 ± 5.7	4.5 ± 0.8 m/4.7 ± 0.9 m	NR	NR	Zhen yuan capsule + duloxetine	Duloxetine	8 w	NR	HAMD scores, cure rate, effective rate, adverse events

Jin and Ying 2013 [40]	DSM-IV	50/50	26 ± 3/27.0 ± 2.5	7 ± 4 m/8 ± 3 m	NR	Strengthening heart and spleen, relieving liver qi stagnation.	Shugan jieyu capsule + paroxetine	Paroxetine	8 w	NR	Effective rate, HAMD scores

Lei and Fu 2015 [41]	CCMD-III, DSM-V	49/49	27.38 ± 5.69/27.68 ± 5.53	NR	NR	NR	Jieyu xiaoyou decoction + amitriptyline	Amitriptyline	6 w	NR	Cure rate, effective rate, EPDS scores, adverse events

Li et al. 2013 [42]	DSM-IV	75/75	25.4 ± 3.6/25.8 ± 3.2	NR	NR	NR	Modification decoction of taohe chengqi and xiao chaihu	Fluoxetine	6 w	NR	EPDS scores, HAMD scores, effective rate, adverse events

Li and Zhang 2014 [43]	DSM-IV	30/30	26.4 ± 6.5/26.4 ± 6.5	NR	NR	NR	Self-made jieyu xiaoyou decoction + fluoxetine	Fluoxetine	4 w	6 m	Effective rate, adverse events

Liang et al. 2012 [44]	DSM-IV	60/60	26.12 ± 4.1/25.74 ± 5.01	22 ± 7.65 d/21 ± 6.73 d	NR	NR	Xiaoyao pill + fluoxetine	Fluoxetine	6 w	NR	Effective rate, HAMD scores, adverse events

Lin et al. 2008 [45]	DSM-IV	37/31	27.14 ± 4.75/26.57 ± 4.16	NR	NR	Relieving liver qi and invigorating the spleen.	Electuary of xiaoyao san + paroxetine	Paroxetine	6 w	NR	Effective rate, obvious effective rate, HAMD scores

Liu 2015 [46]	CCMD-III	43/39	29.23 ± 8.91/28.39 ± 8.31	NR	Deficiency of qi and blood	NR	Huo li su oral liquid + sertraline hydrochloride	Sertraline hydrochloride	8 w	NR	HAMD scores, aerum norepinephrine, aerum 5-hydroxytryptamine, effective rate, adverse events

Lv 2007 [47]	DSM-IV	30/30	24.9 ± 2.1/25.2 ± 1.8	NR	Deficiency of both heart and spleen	Strengthening heart and spleen.	Gui pi decoction + supportive psychotherapy	Supportive psychotherapy	4 w	NR	Effective rate, HAMD scores, estradiol, progesterone

Mi et al. 2014 [48]	DSM-IV	43/43	31.23 ± 5.33/31.48 ± 5.81	10.19 ± 2.98 d/11.09 ± 2.01 d	Liver depression and spleen deficiency	Invigorating the spleen and regulating the liver.	Decoction of jianpi tiaogan + fluoxetine hydrochloride dispersible tablets	Fluoxetine hydrochloride dispersible tablets	4 w	NR	Effective rate, EPDS scores

Pan and Wu 2013 [49]	CCMD-III	30/30	26.23 ± 33.45/25.68 ± 35.26	1.56 ± 1.25 m/1.45 ± 1.50 m	NR	NR	Pill of danzi xiaoyao san + Venlafaxine	Venlafaxine	12 w	24 w	Obvious effective rate, effective rate, HAMD scores, adverse events

Qian 2014 [50]	DSM-IV	39/38	25.7 ± 4.5/25.3 ± 4.4	4.2 ± 2.3 m/4.3 ± 2.5 m	NR	NR	Shugan jieyu capsule	Paroxetine	8 w	NR	HAMD scores, effective rate, adverse events

Ran 2013 [21]	DSM-IV	42/40	30.26 ± 3.57/31.26 ± 4.05	NR	NR	NR	Decoction of danzhi xiaoyao san + venlafaxine hydrochloride sr capsules + health and physical education + psychological counseling	Venlafaxine hydrochloride sr capsules + health and physical education + psychological counseling	12 w	NR	HAMD scores, clinical control rate, effective rate

Ren 2009 [51]	DSM-IV	30/30	25.6/26.1	12.5 d/12.1 d	NR	NR	Modification decoction of xiaoyao san + Fluoxetine	Fluoxetine	6 w	NR	Effective rate, adverse events

Shao et al. 2011 [52]	DSM-IV	50/50	28.1/28.4	29.5 d/37.3 d	NR	Relieving liver qi stagnation, tranquilizing the mind, regulating spleen and stomach.	Xiao chaihu decoction + paroxetine	Paroxetine	30 d	NR	Effective rate, HAMD scores, adverse events

Shi 2013 [53]	DSM-IV	40/36	29/29	NR	NR	NR	Self-made liqi yangxue decoction + Fluoxetine hydrochloride	Fluoxetine hydrochloride	4 w	NR	Effective rate, adverse events

Su 2014 [54]	DSM-IV	59/29	21.8/25.4	22.5 d/24.3 d	NR	Nourishing the blood and tranquilizing mind, regulate qi removing stagnation.	Shugan jieyu decoction + paroxetine	Paroxetine	6 w	NR	Cure rate, HAMD scores, adverse events

Sun 2012 [55]	DSM-IV	32/33	23.7 ± 2.6/23.4 ± 2.7	127.4 ± 33.7 d/129.3 ± 34.6 d	NR	Relieving liver qi stagnation, blood activating and stasis dissolving, regulating psychiatry.	Modification decoction of xiaoyao san + fluoxetine	Fluoxetine	3 m	NR	HAMD scores, obvious effective rate, effective rate, adverse events

Wang et al. 2011 [56]	DSM-IV	27/29	29.2 ± 1.6/29.4 ± 1.9	4.6 ± 1.4 m/4.5 ± 1.2 m	NR	Enriching blood, stabling qi, relieving qi stagnation.	Self-made rougan decoction + fluoxetine	Fluoxetine	4 w	NR	Effective rate, adverse events

Wang 2012 [57]	DSM-IV	30/30	30.07 ± 3.3/30.43 ± 3.43	NR	Deficiency of both heart and spleen	Strengthening heart and spleen.	Shenqi jieyu granules	Placebo	6 w	NR	Effective rate, clinical control rate, EPDS scores, Chinese medicine symptom score, estradiol, progesterone, adverse events

Wang et al. 2015 [58]	DSM-IV	46/46	27.3 ± 4.6/28.1 ± 4.1	NR	Stagnation of liver qi	NR	Modification mixture of self-made jieyu rougan decoction + mirtazapine + vitamin D drops + psychological counseling	Mirtazapine + vitamin D drops + psychological counseling	6 w	NR	Effective rate, HAMD scores

Wang et al. 2014 [59]	DSM-IV	31/32	31.23 ± 2.22/32.01 ± 1.79	9.39 ± 4.28 d/9.03 ± 3.69 d	NR	NR	Wu ling capsule + sertraline hydrochloride	Sertraline hydrochloride	4 w	NR	Effective rate, estradiol, progesterone, serum OFQ level, serum 5-hydroxytryptamine, adverse events

Wei et al. 2009 [60]	CCMD-III	32/32	27.38 ± 5.135/26.72 ± 4.658	27.03 ± 11.956 d/26.09 ± 9.663 d	Both of liver depression and spleen deficiency and stagnant heat	NR	Xiaoyao pill + sertraline	Sertraline	6 w	NR	Obvious effective rate, effective rate, HAMD scores, adverse events

Wu et al. 2014 [61]	DSM-IV	35/35	23.7 ± 2.6/24.5 ± 2.7	NR	NR	Relieving liver qi stagnation, blood activating and stasis dissolving, regulating psychiatry.	Xiaoyao powder + Sertraline	Sertraline	3 m	NR	HAMD scores, obvious effective rate, effective rate, adverse events

Xu et al. 2006 [62]	DSM-IV	30/15	27.6/27.4	38.6 d/37.3 d	NR	NR	Bu xin pill	Paroxetine	3–6 m	NR	Effective rate, cure rate, adverse events

Xu et al. 2013 [63]	DSM-IV	50/50	30.43 ± 3.43/30.07 ± 3.34	NR	Kidney deficiency and liver stagnation	Nourishing kidney deficiency, regulating qi and tranquilizing the mind.	Yinao jieyu fang granule	Placebo	6 w	NR	Effective rate, estradiol, progesterone, adverse events

Zhang et al. 2014 [64]	ICD-X	48/48	27.03 ± 5.12/26.72 ± 4.67	27.38 ± 11.95 d/26.09 ± 9.66 d	NR	NR	Shugan jieyu capsule	Fluoxetine	6 w	NR	Effective rate, HAMD scores, adverse events

Zhang 2015 [65]	CCMD-III	48/48	26.8 ± 4.3/25.9 ± 4.8	4.3 ± 0.7 m/4.1 ± 0.8 m	NR	NR	Shugan jieyu capsule	Citalopram	6 w	NR	HAMD scores, effective rate, adverse events

Zhang and Liu 2009 [66]	DSM-IV	46/42	29.04 ± 3.9/29.12 ± 4.26	38.2 d/41.7 d	NR	NR	Wu ling capsule + fluoxetine + psychological therapy	Fluoxetine + psychological therapy	6 w	NR	HAMD scores, effective rate, adverse events

Zhao and Lin 2006 [67]	DSM-IV	45/42	29.04 ± 3.99/29.12 ± 4.26	NR	NR	Normalizing qi dynamic	Decoction of chaihu shugan san + fluoxetine	Fluoxetine	4 w	NR	HAMD scores, effective rate, adverse events

Zheng 2013 [68]	DSM-IV	54/54	30.44 ± 5.2/30.56 ± 5.13	10.28 ± 3.35 d/9.48 ± 3.35 d	Deficiency of qi and blood and blood stasis	Strengthening primordial qi, activating and nourishing blood, tranquilize the mind.	Shen gui ren mixture + psychological counseling	Psychological counseling	6 w	NR	Effective rate, EPDS scores, Chinese medicine symptom score, adverse events

Zheng 2015 [26]	DSM-IV	39/39	28.6 ± 4.1/29.0 ± 4.3	4.2 ± 0.5 m/4.0 ± 0.4 m	NR	Relieving liver qi stagnation, blood-activating and stasis-dissolving, d regulating psychiatry.	Decoction of shenghua xiaoyao san + paroxetine	Paroxetine	8 w	NR	Effective rate, adverse events

Zhou 2013 [69]	CCMD-III	25/25	22–32/22–32	NR	NR	NR	Self-made yangxue ningshen decoction + sertraline	Sertraline	40 d	NR	Effective rate, HAMD scores, adverse events

Zhou et al. 2015 [70]	DSM-IV	30/30	26.15 ± 2.6/25.85 ± 2.9	NR	NR	NR	Modification mixture of yueju wan and ganmai dazao tang + psychological therapy	Psychological therapy	4 w	NR	HAMD scores, effective rate

Zhu 2008 [71]	DSM-IV	50/50	28.1/28.4	25.9 d/37.3 d	NR	NR	Yangxin jieyu decoction	Paroxetine	30 d	NR	Effective rate, HAMD scores, adverse events

Zhu 2014 [72]	DSM-IV	32/31	23.32 ± 1.86/23.42 ± 2.06	NR	NR	Relieving liver qi stagnation, enriching blood and promoting blood flow, enriching blood to anchor the mind, clearing heat and relieving fidgetiness.	Modification mixture of suan zao ren decoction	Paroxetine	4 w	NR	Effective rate, HAMD scores, adverse events

I: intervention group; C: control group; TCM: traditional Chinese medicine; DSM: diagnostic and statistical manual of mental disorders; CCMD: Chinese classification of mental disorders; ICD: international classification of diseases; NR: not reported; d: day; w: week; m: month.

**Table 2:** Composition of formula and categories of TCM therapeutic methods.

Study ID	Categories of TCM therapeutical methods	Formula	Composition of formula
Cai 2015 [28]	MRLQS + MSHS	Shugan jianpi kaixin decoction	Radix bupleuri, Rhizoma chuanxiong, Rhizoma cyperi rotundi, Pericarpium citri reticulatae, Radix paeoniae alba, Radix astragali, Rhizoma atractylodes macrocephalae, Radix angelicae sinensis, Radix polygalae tenuifoliae, Semen ziziphi spinosae, Fructus jujubae.

Chen et al. 2011 [30]	MRLQS	Self-made qingyu pingxin decoction	Massa medica fermentata, Radix bupleuri, Rhizoma cyperi rotundi, Rhizoma atractylodes macrocephalae, Radix paeoniae alba, Radix rehmanniae, Tu ophiopogonis japonici, Radix et rhizoma rhei, Radix glycyrrhizae.

Chen et al. 2015 [29]	MRLQS + MSHS	Fuling shenzhi shuangxin pill	Poria, Sclerotium pararadicis poriae cocos, Cinnabaris, Radix polygalae tenuifoliae, Radix panacis ginseng, Semen ziziphi spinosae, Radix bupleuri, Radix glycyrrhizae.

Ding et al. 2010 [31]	MRLQS	Modification decoction of xiaoyao san	Radix bupleuri, Radix paeoniae alba, Radix angelicae sinensis, Rhizoma atractylodes macrocephalae, Poria, Tuber curcumae, cortex albizziae julibrissinis, Radix polygalae tenuifoliae, Semen ziziphi spinosae, Rhizoma cyperi rotundi, Radix glycyrrhizae.

Fan 2011 [32]	MRLQS + MTQB	Modification mixture of tianwang buxin decoction	Tuber curcumae, Fructus schisandrae chinensis, Semen biotae orientalis, Radix salviae miltiorrhizae, Radix paeoniae alba, Radix rehmanniae, Radix polygalae tenuifoliae, Radix platycodi grandiflori, Radix bupleuri, Cucurbita pepo, Semen ziziphi spinosae, Radix codonopsitis pilosulae, Poria, Radix angelicae sinensis.

Fang 2014 [33]	MRLQS + MBASD	Self-made tiaoxue jieyu decoction	Radix bupleuri, Radix angelicae sinensis, Radix paeoniae alba, Poria, Cortex magnoliae officinalis, Rhizoma cyperi rotundi, Tuber curcumae, Cortex albizziae julibrissinis, Semen nelumbinis nucifarae, Semen ziziphi spinosae, Herba leonuri, Radix pseudoginseng, Fructus crataegi, Herba lophatheri, Radix polygalae tenuifoliae, Rhizoma acori tatarinowii, Radix glycyrrhizae.

Gao 2010 [34]	MTQB	Modification mixture of yulin decoction	Cornu degelatinum cervi, Semen cuscutae chinensis, Radix rehmanniae, Radix angelicae sinensis, Radix paeoniae alba, Rhizoma chuanxiong, Radix codonopsitis pilosulae, Rhizoma atractylodes macrocephalae, Poria, Cortex eucommiae, Radix morindae officinalis, Rhizoma cyperi rotundi, Radix glycyrrhizae.

Guo et al. 2011 [35]	MRLQS + MSHS	Modification decoction of chaihu shugan san and ganmai dazao tang	Radix bupleuri, Fructus citri aurantii, Rhizoma cyperi rotundi, Pericarpium citri reticulatae, Radix paeoniae alba, Rhizoma chuanxiong, Radix glycyrrhizae, Fructus levis tritici aestiva, Fructus jujubae.

Hao 2015 [36]	MRLQS + MSHS	Shugan jieyu capsule	Hypericum perforatum L., Acanthopanax senticosus.

He and Ren 2008 [37]	MRLQS	Modification decoction of xiaoyao san	Radix bupleuri, Poria, Rhizoma atractylodis macrocephalae, Rhizoma cyperi rotundi, Radix rehmanniae, Radix paeoniae alba, Radix et rhizoma rhei, Radix glycyrrhizae.

Hu 2013 [38]	MRLQS	Xiaoyao pill	Radix angelicae sinensis, Radix paeoniae alba, Rhizoma atractylodes macrocephalae, Radix bupleuri, Poria, and so forth.

Jiang et al. 2012 [39]	MTQB	Zhen yuan capsule	Main flavour components is ginseng fruit saponins.

Jin and Ying 2013 [40]	MRLQS + MSHS	Shugan jieyu capsule	Hypericum perforatum L, Acanthopanax senticosus.

Lei and Fu 2015 [41]	MRLQS + MSHS	Jieyu xiaoyou decoction	Radix bupleuri, Rhizoma cyperi rotundi, Radix paeoniae alba, Radix panacis ginseng, Poria, Sclerotium pararadicis poriae cocos, Radix angelicae sinensis, Rhizoma atractylodes macrocephalae, Radix polygalae tenuifoliae, Radix glycyrrhizae, Tu ophiopogonis japonici, Tuber curcumae.

Li et al. 2013 [42]	MRLQS + MSHS + MBASD	Modification decoction of taohe chengqi and xiao chaihu	Semen pruni persicae, Radix cinnamomi cassiae, Rheum officinale Baill, Radix bupleuri, Radix scutellariae baicalensis, Pinellia ternata, Rhizoma cyperi rotundi, Radix panacis ginseng, Kadsura interior, Ficus simplicissima lour, Tuber curcumae, Os draconis, Semen ziziphi spinosae, Radix glycyrrhizae.

Li and Zhang 2014 [43]	MRLQS	Self-made jieyu xiaoyou decoction	Radix bupleuri, Poria, Radix paeoniae alba, Rhizoma atractylodes macrocephalae, Radix angelicae sinensis, Rhizoma cyperi rotundi, Radix polygalae tenuifoliae, Tuber curcumae, Tu ophiopogonis japonici.

Liang et al. 2012 [44]	MRLQS	Xiaoyao pill	Radix angelicae sinensis, Radix paeoniae alba, Radix bupleuri, Rhizoma atractylodes macrocephalae, Radix glycyrrhizae, Poria, Rhizoma zingiberis preparatum, Peppermint.

Lin et al. 2008 [45]	MRLQS	Electuary of xiaoyao san	Radix bupleuri, Radix paeoniae alba, Radix angelicae sinensis, Poria, Rhizoma atractylodes macrocephalae, Radix glycyrrhizae, Peppermint, Uncooked rhizoma zingiberis.

Liu 2015 [46]	MTQB	Huo li su oral liquid	Radix astragali, Rhizoma polygonati, Fructus lycii chinensis, Radix polygoni multiflori, Radix salviae miltiorrhizae, Herba epimedii.

Lv 2007 [47]	MSHS	Gui pi decoction	Radix astragali, Arillus longan, Radix panacis ginseng, Rhizoma atractylodes macrocephalae, Radix angelicae sinensis, Sclerotium pararadicis poriae cocos, Semen ziziphi spinosae, Radix polygalae tenuifolia, Radix aucklandiae, Radix glycyrrhizae.

Mi et al. 2014 [48]	MRLQS + MSHS	Decoction of jianpi tiaogan	Radix astragali, Radix angelicae sinensis, Radix paeoniae alba, Radix bupleuri, Poria, Rhizoma atractylodes macrocephalae, Rhizoma pinelliae ternatae, Cortex magnoliae officinalis, Rhizoma polygonati, Uncooked rhizoma zingiberis, Caulis perillae, Radix glycyrrhizae.

Pan and Wu 2013 [49]	MRLQS	Danzi xiaoyao pill	Radix bupleuri, Radix angelicae sinensis, Radix paeoniae alba, Poria, Rhizoma atractylodes macrocephala, Cortex radix moutan, Fructus gardeniae jasminoidis, Peppermint, Radix glycyrrhizae.

Qian 2014 [50]	MRLQS + MSHS	Shugan jieyu capsule	Hypericum perforatum L, Acanthopanax senticosus.

Ran 2013 [21]	MRLQS	Decoction of danzhi xiaoyao san	Radix bupleuri, Peppermint, Cortex radix moutan, Fructus gardenige, Radix angelicae sinensis, Radix paeoniae alba, Rhizoma atractylodes macrocephalae, Poria, Radix glycyrrhizae.

Ren 2009 [51]	MRLQS	Modification decoction of xiaoyao san	Radix bupleuri, Poria, Radix paeoniae alba, Rhizoma atractylodes macrocephalae, Radix angelicae sinensis, Radix et rhizoma rhei, Rhizoma cyperi rotundi, Tu ophiopogonis japonici, Radix rehmanniae.

Shao et al. 2011 [52]	MRLQS	Xiao chaihu decoction	Radix bupleuri, Radix scutellariae baicalensis, Rhizoma pinelliae ternatae, Fructus jujubae, Radix glycyrrhizae, Uncooked rhizoma zingiberis, Radix paeoniae alba, Rhizoma atractylodes macrocephalae, Bulbus lilii, Semen ziziphi spinosae.

Shi 2013 [53]	MRLQS + MSHS	Self-made liqi yangxue decoction	Radix bupleuri, Radix angelicae sinensis, Fructus citri aurantii, Rhizoma cyperi rotundi, Pericarpium citri reticulatae, Tuber curcumae, Radix paeoniae alba, Radix astragali, Radix codonopsitis pilosulae, Fructus jujubae, Arillus longan, Sclerotium pararadicis poriae cocos, Semen biotae orientalis, Radix polygalae tenuifoliae, Semen nelumbinis nucifarae, Radix glycyrrhizae.

Su 2014 [54]	MRLQS + MBASD	Shugan jieyu decoction	Radix bupleuri, Radix angelicae sinensis, Radix paeoniae alba, Tuber curcumae, Poria, Rhizoma chuanxiong, Rhizoma cyperi rotundi, cortex albizziae julibrissinis, Flos carthami tinctorii, Semen pruni persicae, Radix rehmanniae, Bulbus lilii.

Sun 2012 [55]	MRLQS	Modification decoction of xiaoyao san	Radix bupleuri, Poria, Radix paeoniae alba, Rhizoma atractylodes macrocephalae, Radix angelicae sinensis, Semen ziziphi spinosae, Rhizoma chuanxiong, Radix glycyrrhizae.

Wang et al. 2011 [56]	MRLQS	Self-made rougan decoction	Radix bupleuri, Radix angelicae sinensis, Rhizoma atractylodes macrocephalae, Radix paeoniae alba, Tuber curcumae, Citrus reticulata blanco, Radix rehmanniae, Tu ophiopogonis japonici, Radix glycyrrhizae.

Wang 2012 [57]	MSHS	Shenqi jieyu granules	Radix astragali, Radix codonopsitis pilosulae, Radix angelicae sinensis, Semen ziziphi spinosae, Fructus corni, and so forth.

Wang et al. 2014 [59]	MTQB	Wu ling capsule	Main flavour components is Xylaria nigripes powder.

Wang et al. 2015 [58]	MRLQS	Modification mixture of self-made jieyu rougan decoction	Radix bupleuri, Fructus citri aurantii, Rhizoma cyperi rotundi, Pericarpium citri reticulatae, Radix angelicae sinensis, Radix paeoniae alba, Cortex albizziae julibrissinis, Tuber fleeceflower stem, Semen ziziphi spinosae, Rhizoma atractylodes macrocephalae, Poria, Uncooked rhizoma zingiberis, Radix glycyrrhizae.

Wei et al. 2009 [60]	MRLQS	Xiaoyao pill	Radix bupleuri, Radix angelicae sinensis, Radix paeoniae alba, Poria, Rhizoma atractylodis macrocephalae, Radix clycyrrhizae, and so forth.

Wu et al. 2014 [61]	MRLQS	Xiaoyao powder	Radix bupleuri, Poria, Radix paeoniae alba, Rhizoma atractylodes macrocephalae, Radix angelicae sinensis, Semen ziziphi spinosae, Rhizoma chuanxiong, Radix glycyrrhizae.

Xu et al. 2006 [62]	MTQB	Bu xin pill	Radix angelicae sinensis, Rhizoma chuanxiong, Radix paeoniae alba, Radix rehmanniae, Radix panacis ginseng, Radix scrophulariae ningpoensis, Tu ophiopogonis japonici, Radix asparagi, Cinnabaris, Poria, Radix polygalae tenuifoliae, Semen ziziphi spinosae, Semen biotae orientalis.

Xu et al. 2013 [63]	MRLQS + MTQB	Yinao jieyu fang granule	Acanthopanax senticosus, Tuber curcumae, and so forth.

Zhang and Liu 2009 [66]	MTQB	Wu ling capsule	Main flavour components is Xylaria nigripes powder.

Zhang et al. 2014 [64]	MRLQS + MSHS	Shugan jieyu capsule	Hypericum perforatum L, Acanthopanax senticosus.

Zhang 2015 [65]	MRLQS + MSHS	Shugan jieyu capsule	Hypericum perforatum L, Acanthopanax senticosus.

Zhao and Lin 2006 [67]	MRLQS	Decoction of chaihu shugan san	Radix bupleuri, Fructus citri aurantii, Rhizoma cyperi rotundi, Pericarpium citri reticulatae, Radix paeoniae alba, Rhizoma chuanxiong, Radix glycyrrhizae.

Zheng 2013 [68]	MTQB + MBASD	Shen gui ren mixture	Radix panacis ginseng, Radix angelicae sinensis, Semen ziziphi spinosae.

Zheng 2015 [26]	MRLQS + MBASD	Decoction of shenghua xiaoyao san	Radix angelicae sinensis, Radix rubrus paeoniae lactiflorae, Poria, Rhizoma atractylodes macrocephalae, Semen pruni persicae, Radix bupleuri, Rhizoma zingiberis preparatum, Peppermint, Radix glycyrrhizae.

Zhou 2013 [69]	MRLQS + MTQB	Self-made yangxue ningshen decoction	Radix angelicae sinensis, Radix paeoniae alba, Radix bupleuri, Radix salviae miltiorrhizae, Tuber curcumae, Semen ziziphi spinosae, cortex albizziae julibrissinis, Radix polygalae tenuifoliae, Radix glycyrrhizae.

Zhou et al. 2015 [70]	MRLQS + MSHS	Modification mixture of yueju wan and ganmai dazao tang	Rhizoma cyperi rotundi, Rhizoma chuanxion, Rhizoma atractylodis, Massa medica fermentata, Fructus gardeniae jasminoidis, Radix glycyrrhizae, Fructus levis tritici aestiva, Fructus jujubae.

Zhu 2008 [71]	MRLQS + MTQB	Yangxin jieyu decoction	Radix astragali, Radix bupleuri, Radix scutellariae baicalensis, Radix panacis ginseng, Radix glycyrrhizae, Radix rehmanniae, Fructus schisandrae chinensis, Radix angelicae sinensis, Semen biotae orientalis, Semen ziziphi spinosae, Radix salviae miltiorrhizae, Uncooked rhizoma zingiberis, Fructus jujubae.

Zhu 2014 [72]	MRLQS + MTQB + MBASD	Modification mixture of suan zao ren decoction	Semen ziziphi spinosae, Poria, Rhizoma chuanxiong, Rhizoma anemarrhenae, Tuber curcumae, Radix angelicae sinensis, Radix glycyrrhizae, Radix bupleuri.

MRLQS: the method of relieving liver qi stagnation (shu gan jie yu fa); MSHS: the method of strengthening heart and spleen (bu yi xin pi fa); MBASD: the method of blood-activating and stasis-dissolving (huo xue hua yu fa); MTQB: the method of tonifying qi and blood (bu yi qi xue fa).

**Table 3:** Effect estimates of CHM for PPD.

Outcome or subgroup	Trials	Effect estimates [95% CI]	*P* value
*(1) HAMD scores*			
*(1.1) CHM *versus* routine treatments*			
MRLQS + MSHS versus routine treatments	4	MD −1.91 [−3.37, −0.44]	0.01
MRLQS + MTQB versus routine treatments	1	MD −7.30 [−8.23, −6.37]	<0.00001
MRLQS + MSHS + MBASD versus routine treatments	1	MD −2.93 [−4.36, −1.50]	<0.0001
MRLQS + MTQB + MBASD versus routine treatments	1	MD −0.22 [−2.40, 1.96]	0.84
* Meta-analysis [REM] (heterogeneity: I* ^*2*^ * = 95%, P < 0.00001)*	7	MD −2.60 [−4.55, −0.64]	0.009
*(1.2) CHM + routine treatments *versus* routine treatments*			
MRLQS + routine treatments versus routine treatments	13	MD −3.19 [−4.41, −1.97]	<0.00001
MSHS + routine treatments versus routine treatments	1	MD −4.10 [−6.23, −1.97]	0.0002
MTQB + routine treatments versus routine treatments	3	MD −2.67 [−4.48, −0.87]	0.004
MRLQS + MSHS + routine treatments versus routine treatments	4	MD −2.67 [−3.20, −2.14]	<0.00001
MRLQS + MBASD + routine treatments versus routine treatments	1	MD −4.10 [−6.65, −1.55]	0.002
MRLQS + MTQB + routine treatments versus routine treatments	1	MD −1.74 [−3.96, 0.48]	0.12
* Meta-analysis [REM] (heterogeneity: I* ^*2*^ * = 85%, P < 0.00001)*	23	MD −3.00 [−3.73, −2.26]	<0.00001
*(2) EPDS scores*			
*(2.1) CHM *versus* placebo*			
MSHS versus placebo	1	MD −2.67 [−3.88, −1.46]	<0.0001
*(2.2) CHM *versus* routine treatments*			
MTQB + MBASD versus routine treatments	1	MD −4.70 [−5.76, −3.64]	<0.00001
MRLQS + MSHS + MBASD versus routine treatments	1	MD −1.95 [−3.30, −0.60]	0.005
* Meta-analysis [REM] (heterogeneity: I* ^*2*^ * = 90%, P = 0.002)*	2	MD −3.36 [−6.05, −0.66]	0.01
*(2.3) CHM + routine treatments *versus* routine treatments*			
MRLQS + MSHS + routine treatments versus routine treatments	3	MD −3.80 [−5.27, −2.34]	<0.00001
* Meta-analysis [REM] (heterogeneity: I* ^*2*^ * = 75%, P = 0.02)*	3	MD −3.80 [−5.27, −2.34]	<0.00001
*(3) Serum estradiol*			
*(3.1) CHM *versus* placebo*			
MSHS versus placebo	1	MD 11.55 [−8.58, 31.68]	0.26
MRLQS + MTQB versus placebo	1	MD 43.85 [34.90, 52.80]	<0.00001
* Meta-analysis [REM] (heterogeneity: I* ^*2*^ * = 88%, P = 0.004)*	2	MD 29.01 [−2.54, 60.56]	0.07
*(3.2) CHM + routine treatments *versus* routine treatments*			
MSHS + routine treatments versus routine treatments	1	MD 26.73 [5.06, 48.40]	0.02
MTQB + routine treatments versus routine treatments	1	MD 38.58 [35.58, 41.58]	<0.00001
* Meta-analysis [FEM] (heterogeneity: I* ^*2*^ * = 11%, P = 0.29)*	2	MD 38.36 [35.38, 41.33]	<0.00001
*(4) Progesterone*			
*(4.1) CHM *versus* placebo*			
MSHS versus placebo	1	MD −1.99 [−7.28, 3.30]	0.46
MRLQS + MTQB versus placebo	1	MD −2.11 [−3.10, −1.12]	<0.0001
* Meta-analysis [FEM] (heterogeneity: I* ^*2*^ * = 0%, P = 0.97)*	2	MD −2.11 [−3.08, −1.14]	<0.0001
*(4.2) CHM + routine treatments *versus* routine treatments*			
MSHS + routine treatments versus routine treatments	1	MD −3.89 [−8.92, 1.14]	0.13
MTQB + routine treatments versus routine treatments	1	MD −11.64 [−13.41, −9.87]	<0.00001
* Meta-analysis [REM] (heterogeneity: I* ^*2*^ * = 88%, P = 0.004)*	2	MD −8.14 [−15.70, −0.58]	0.03
*(5) Incidence of adverse events*			
*(5.1) CHM versus placebo*			
MSHS versus placebo	1	RR 0.20 [0.01, 4.00]	0.29
MRLQS + MTQB versus placebo	1	RR 3.00 [0.13, 71.92]	0.50
* Meta-analysis [FEM] (heterogeneity: I* ^*2*^ * = 33%, P = 0.22)*	2	RR 0.67 [0.11, 3.91]	0.65
*(5.2) CHM *versus* routine treatments*			
MTQB versus routine treatments	2	RR 0.12 [0.04, 0.36]	0.0001
MRLQS + MSHS versus routine treatments	4	RR 0.21 [0.10, 0.47]	0.0001
MRLQS + MTQB versus routine treatments	1	RR 0.06 [0.00, 0.99]	0.05
MRLQS + MSHS + MBASD versus routine treatments	1	RR 0.73 [0.31, 1.71]	0.46
MRLQS + MTQB + MBASD versus routine treatments	1	RR 0.03 [0.00, 0.42]	0.010
* Meta-analysis [REM] (heterogeneity: I* ^*2*^ * = 57%, P = 0.02) *	9	RR 0.18 [0.09, 0.38]	<0.00001
*(5.3) CHM + routine treatments *versus* routine treatments*			
MRLQS + routine treatments versus routine treatments	9	RR 0.45 [0.32, 0.64]	<0.00001
MTQB + routine treatments versus routine treatments	3	RR 0.76 [0.31, 1.84]	0.54
MRLQS + MSHS + routine treatments versus routine treatments	3	RR 0.45 [0.22, 0.93]	0.03
MRLQS + MTQB + routine treatments versus routine treatments	2	RR 0.32 [0.09, 1.14]	0.08
MRLQS + MBASD + routine treatments versus routine treatments	2	RR 0.73 [0.18, 2.93]	0.66
* Meta-analysis [REM] (heterogeneity: I* ^*2*^ * = 56%, P = 0.002)*	19	RR 0.49 [0.37, 0.65]	<0.00001
*(6) TESS*			
*(6.1) CHM *versus* routine treatments*			
MRLQS + MSHS versus routine treatments	1	MD −2.05 [−3.00, −1.10]	<0.0001
*(6.2) CHM + routine treatments *versus* routine treatments*			
MRLQS + routine treatments versus routine treatments	2	MD −2.31 [−3.12, −1.50]	<0.00001
MTQB + routine treatments versus routine treatments	1	MD −3.02 [−4.25, −1.79]	<0.00001
MRLQS + MTQB + routine treatments versus routine treatments	1	MD −0.83 [−1.50, −0.16]	0.01
MRLQS + MBASD + routine treatments versus routine treatments	1	MD −1.40 [−1.72, −1.08]	<0.00001
* Meta-analysis [REM] (heterogeneity: I* ^*2*^ * = 71%, P = 0.008)*	5	MD −1.80 [−2.46, −1.14]	<0.00001
*(7) SERS*			
*(7.1) CHM + routine treatments *versus* routine treatments*			
MRLQS + routine treatments versus routine treatments	2	MD −5.19 [−9.73, −0.64]	0.03
* Meta-analysis [REM] (heterogeneity: I* ^*2*^ * = 98%, P < 0.00001)*	2	MD 5.19 [−9.73, −0.64]	0.03

FEM: fixed effects model, REM: random effects model.
