# Supplementary material for: Chinese Herbal Medicine for Postpartum Depression: A Systematic Review of Randomized Controlled Trials
Source: Evid Based Complement Alternat Med. 2016 Sep 28;2016:5284234. doi: 10.1155/2016/5284234 (PMC5059536; doi:10.1155/2016/5284234)
Supplement: Supplementary file 1 — Details regarding search strategies of databases and risk of bias summary are shown in supplementary material. Search terms used in each database was included in supplementary Table 1. Review authors' judgments about each risk of bias item for included studies were shown in supplementary Figure 1. [file 5284234.f1.pdf]

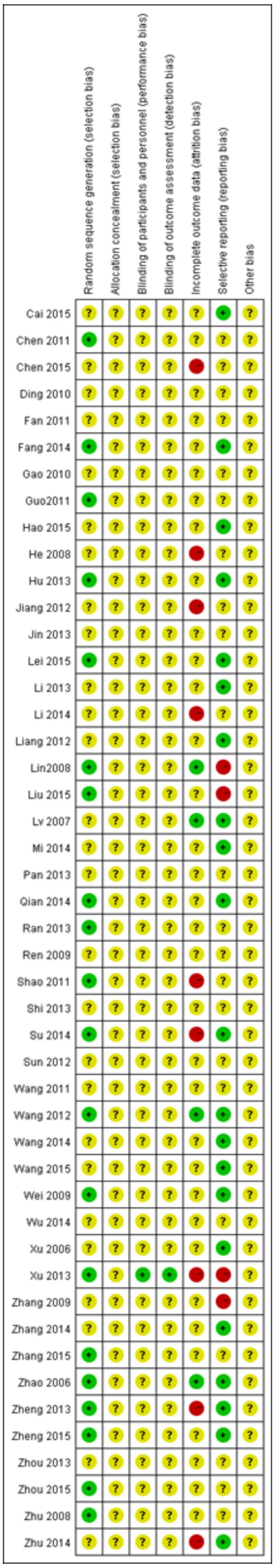

Supplementary Fig 1: Risk of bias summary: review authors' judgements about each risk of bias item for included study. Red (-): high risk of bias; Yellow (?): unclear risk; Green (+): low risk of bias

Supplementary Table 1: Search terms used in databases

Datebases	Search terms
CNKI	发表时间 between (1900-1-1,2015-12-31 and 摘要=产后 or 摘要=产妇 or 摘要=分娩 or 摘要=剖宫产 or 摘要=产褥期 and 全文=抑郁 and 全文=中医 or 全文=中药 or 全文=中西医 or 全文=草药 or 全文=汤药) (精确匹配)
CBM	(((((("产后"[摘要:智能]) OR "产妇"[摘要:智能]) OR "分娩"[摘要:智能]) OR "剖宫产"[摘要:智能]) OR "产褥期"[摘要:智能]) AND -2015[日期]) AND ("抑郁"[摘要:智能]) AND -2015[日期]
VIP	文摘=产后 或者 文摘=产妇 或者 文摘=分娩 或者 文摘=剖宫产 或者 文摘=产褥期 并且 年份=1989-2015 并且 文摘=抑郁 并且 年份=1989-2015
Wan Fang	摘要:(产妇 + 产后+ 分娩 + 剖宫产 + 产褥期) * 摘要:(抑郁) * Date:-2015
PubMed	((depression, postpartum[MeSH Terms]) AND (((((Chinese traditional) OR Chinese herb*) OR Oriental traditional) OR herb) OR herbal medicine)) AND random* AND (("0001/01/01"[PDat] : "2015/12/31"[PDat]))
EMBASE	#1 maternal AND ('depression'/exp OR depression)<br>#2 post AND partum AND ('depression'/exp OR depression)<br>#3 postnatal AND ('depression'/exp OR depression)<br>#4 postpartum AND ('depression'/exp OR depression)<br>#5 'puerperium'/exp OR puerperium AND ('depression'/exp OR depression)<br>#6 #1 OR #2 OR #3 OR #4 OR #5<br>#7 herbal AND medicine<br>#9 oriental AND traditional<br>#10 Chinese AND herb*<br>#11 Chinese AND traditional<br>#12 #7 OR #8 OR #9 OR #10 OR #11<br>#13 random*<br>#14 #6 AND #12 AND #13

Supplementary Table 1: Continued

CENTRAL	maternal depression or post partum depression or postpartum depression or puerperium depression or postnatal depression in Title, Abstract, Keywords and Chinese traditional or Chinese herb or Oriental traditional or herb or herbal medicine and random , Publication Year to 2015
ICTRP portal	#1 maternal depression OR post partum depression OR postpartum depression OR puerperium depression OR postnatal depression [in the title]<br>#2 Chinese traditional OR Chinese herb* OR Oriental traditional OR herb OR herbal medicine [in the Intervention]<br>#3 #1 AND #2
the website of International Clinical Trial Registry by U.S. National Institutes of Health	Chinese traditional OR Chinese herb* OR Oriental traditional OR herb OR herbal medicine | maternal depression OR post partum depression OR postpartum depression OR puerperium depression OR postnatal depression | received on or before 12/31/2015 | updated on or before 12/31/2015
